# Bradycardia during Induction Therapy with All-*trans* Retinoic Acid in Patients with Acute Promyelocytic Leukemia: Case Report and Literature Review

**DOI:** 10.1155/2018/4938797

**Published:** 2018-06-07

**Authors:** Pin-Zi Chen, Yee-Jen Wu, Chien-Chih Wu, Yu-Wen Wang

**Affiliations:** Department of Pharmacy, National Taiwan University Hospital, College of Medicine, National Taiwan University, 7 Chung Shan S. Rd., Taipei, Taiwan

## Abstract

A 41-year-old man with newly diagnosed acute promyelocytic leukemia (APL) received induction chemotherapy, containing all-*trans* retinoic acid (ATRA), idarubicin, and arsenic trioxide. On the 11th day of therapy, he experienced complete atrioventricular (AV) block; therefore, ATRA and arsenic trioxide were immediately postponed. His heart rate partially recovered, and ATRA was rechallenged with a half dose. However, complete AV block as well as differentiation syndrome recurred on the next day. ATRA was immediately discontinued, and a temporary pacemaker was inserted. Two days after discontinuing ATRA, AV block gradually improved, and ATRA was uneventfully rechallenged again. The Naranjo adverse drug reaction probability scale was 7 for ATRA, suggesting it was the probable cause of arrhythmia. A literature search identified 6 other cases of bradycardia during ATRA therapy, and all of them occurred during APL induction therapy, with onset ranging from 4 days to 25 days. Therefore, monitoring vital signs and performing electrocardiogram are highly recommended during the first month of induction therapy with ATRA. ATRA should be discontinued if complete AV block occurs. Rechallenging with ATRA can be considered in fully recovered and clinically stable patients.

## 1. Introduction

Acute promyelocytic leukemia (APL) is a subtype of acute myeloid leukemia (AML) with distinct morphology, clinical presentation, and cytogenetics. It is characterized by accumulation of abnormal promyelocytes in the bone marrow, potentially fatal coagulopathy, and translocation between chromosomes 15 and 17 [*t*(15;17)] [1, 2].

*t*(15;17) leads to the promyelocytic leukemia and retinoic receptor-*α* fusion gene (PML-RARα), which blocks the differentiation of myeloid cells at the promyelocytic stage. All-*trans* retinoic acid (ATRA or tretinoin) induces a configuration change of PML-RAR*α* or its degradation. As a result of this treatment, abnormal promyelocytes can differentiate into mature myeloid cells, and coagulopathy is significantly reduced [1]. When ATRA combines with anthracycline-based chemotherapy, the complete remission rate is 90%–95%. Therefore, this approach is the standard induction therapy for newly diagnosed APL [1–3].

Arsenic trioxide (ATO) also promotes differentiation of APL cells by targeting the PML moiety and can be used as a synergistic therapy with ATRA [1, 4]. However, rapid differentiation of promyelocytes induced by ATRA or ATO may lead to leukocytosis and differentiation syndrome (DS), especially in patients with an initial white blood cell (WBC) count greater than 10,000/mm^3^. DS is characterized by fever, dyspnea, respiratory distress, pulmonary infiltrates, and pleural or pericardial effusions. This condition is potentially life-threatening; therefore, when DS is suspected, it is crucial to immediately initiate high-dose steroid treatment and temporarily discontinue ATRA and/or ATO in severe DS [2, 5, 6]. Concurrent use of anthracycline-based chemotherapy can decrease the incidence of DS [1, 5, 6].

Other common adverse effects of ATRA include headache, dry skin, xerostomia, bone pain, and nausea [7]. Cardiovascular toxicities, such as arrhythmia, have rarely been reported and are often overlooked by clinical practitioners [8]. Here we report the clinical course of a patient with APL suffering from complete atrioventricular (AV) block during induction therapy with an ATRA-based regimen. Moreover, a literature review was conducted to summarize the clinical characteristics of ATRA-associated arrhythmia.

## 2. Case Report

**Figure 1:**
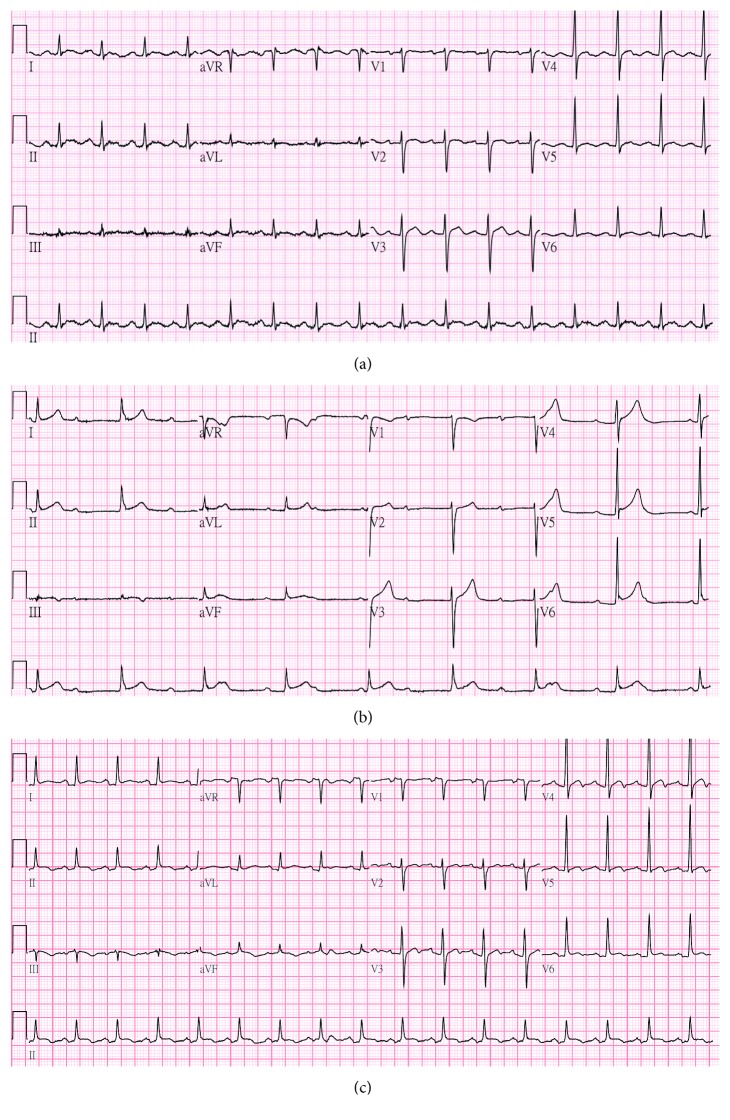
Serial electrocardiogram changes. (a) Normal sinus rhythm before the induction therapy (PR interval 206 ms, QTc 429 ms). (b) The 11th day of therapy, showing complete AV block (PR interval > 400 ms, QTc 395 ms). (c) Restoration of normal sinus rhythm after pacemaker removal (PR interval 192 ms, QTc 353 ms).

A 41-year-old man without any underlying diseases such as cardiovascular disease was hospitalized for spontaneous gum bleeding, epistaxis, and lower limb ecchymosis. Laboratory data on the admission date showed leukocytosis (WBC, 15,820/mm^3^; promyelocyte, 66%, with Auer rod), anemia (Hb, 9.5 g/dL), thrombocytopenia (PLT, 22,000/mm^3^), and abnormal coagulation profile (fibrinogen, 29 mg/dL; fibrin degradation product (FDP), 68.5 mcg/mL; d-dimer, 19.81 mcg/mL; prothrombin time (PT), 20.5 sec; international normalized ratio (INR), 1.92; partial thromboplastin time (PTT), 29 sec). Other laboratory data were as follows: C-reactive protein (CRP), 8.97 mg/dL; total bilirubin (T-bil), 0.73 mg/dL; aspartate aminotransferase (AST), 60 U/L; alanine transferase (ALT), 100 U/L; and serum creatinine (Scr), 0.9 mg/dL. His baseline electrocardiogram (ECG) was normal (Figure 1(a)). Bone marrow aspiration and biopsy disclosed APL with PML-RAR*α*. ATRA at a dose of 45 mg/m^2^/day (40 mg twice daily) was administered. On the third day of therapy, oxygen saturation abruptly dropped to 90% without oxygen supplementation. Chest X-ray (CXR), ECG, and echocardiography did not show any abnormalities. In order to prevent DS, intravenous methylprednisolone was administered at a daily dose of 80 mg–120 mg according to the clinical signs and symptoms. Idarubicin (12 mg/m^2^/dose) was administered starting on the fourth day for four doses. WBC progressively elevated to 46,830/mm^3^; therefore, 1000 mg hydroxyurea twice daily was also added starting on the seventh day.

On the 11th day of therapy, WBC and promyelocyte decreased to 6,310/mm^3^ and 14%, respectively. ATO infusion at a dose of 0.15 mg/kg was initiated; however, dizziness and chest pain occurred during infusion. ECG showed complete AV block with a heart rate (HR) of 40–50 beats/min (bpm) (Figure 1(b)). The electrolytes and liver function tests were all within normal limits (Na, 138 mmol/L; K, 4.6 mmol/L; Ca, 2.29 mmol/L; Mg, 0.95 mmol/L; T-bil, 0.77 mg/dL; AST, 15 U/L; ALT, 14 U/L). Thus, ATO and ATRA were immediately discontinued, and aminophylline was administered.

After interruption of ATRA for three doses, complete AV block persisted, but his HR partially recovered to 60 bpm. ATRA was rechallenged with 40 mg once daily on the 13th day of therapy under relatively stable vital signs. On the following morning, his HR decreased to 30 bpm and he required a nonrebreathing mask to maintain oxygen saturation. CXR showed bilateral pulmonary edema, and echocardiogram revealed a large amount of pericardial effusion with signs of cardiac tamponade. In addition, ECG showed complete AV block with QRS widening, and acute renal failure also occurred (Scr, 1.4 mg/dL; blood urea nitrogen (BUN), 45.2 mg/dL; Na, 133 mmol/L; K, 5.2 mmol/L; Ca, 2.22 mmol/L; Mg, 0.93 mmol/L; P, 4.9 mg/dL; T-bil, 1.22 mg/dL; ALT, 17 U/L). Therefore, ARTA was immediately postponed, and a temporary pacemaker was inserted. Steroid treatment was switched to dexamethasone (8 mg twice daily) because of suspected DS. Pericardiocentesis and thoracentesis were performed. Two days after stopping ATRA, ECG showed sinus bradycardia with a first-degree AV block and diffuse ST elevation. The Naranjo adverse drug reaction probability scale [9] indicated a score of 7 for ATRA, suggesting it was the probable cause of arrhythmia in this episode.

The PML-RAR*α* mutant was still detected using polymerase chain reaction (PCR) of blood sampled on the 19th day of therapy. Consequently, ATRA was resumed the following day with 10 mg twice daily. His HR decreased from 70–90 bpm to 60–70 bpm with stable vital signs. ECG demonstrated sinus rhythm with first-degree AV block. The dose of ATRA was gradually titrated up to 30 mg twice daily on the 24th day. Sinus tachycardia with occasional premature ventricular complexes was noted. The pacemaker was removed on the 27th day. During follow-up, ECG showed normal sinus rhythm (Figure 1(c)), and echocardiogram revealed minimal pericardial effusion.

Complete molecular remission was documented by bone marrow aspiration done on the 38th day of therapy using flow cytometry and PCR for PML-RAR*α*. Thereafter, the patient received three cycles of consolidation with ATRA-based chemotherapy. No further bradycardia episode was reported. The patient has been free of leukemia for 1.5 years after the diagnosis.

## 3. Discussion

The major etiologies of AV block include ischemic heart disease, cardiomyopathy, hyperkalemia, hypothyroidism or hyperthyroidism, and medications. This 41-year-old man did not have any underlying disease, and his baseline CXR, ECG, and echocardiography did not show any abnormalities. The serum potassium level was normal during the first-episode of complete AV block, and it was only mildly elevated and was corrected soon during the second episode of complete AV block. Although idarubicin and ATO have also been reported to cause arrhythmia in the literature, the second arrhythmia episode in this patient recurred only after ATRA rechallenge. Accordingly, ATRA was the most likely culprit drug for this adverse event.

The most common adverse effects of ATRA are headache, dry skin, xerostomia, bone pain, and nausea [7]. Arrhythmia is rarely reported. Therefore, we reported a case of this rare adverse reaction of ATRA and reviewed the literature for other cases. We searched literature reports on PubMed by using the following keywords: (“tretinoin” or “all-*trans* retinoic acid” or “ATRA”) and (“bradycardia” or “tachycardia” or “arrhythmia”). Six other cases of bradycardia during ATRA induction therapy were found and summarized, along with the case reported here, in Table 1 [10–15].

Median onset of bradycardia among the seven patients was 9 days (4–25 days). ECG abnormalities consisted of AV block, sinus bradycardia, and junctional bradycardia [10–15]. Arrhythmia resolved in two patients without interruption of ATRA [12, 14]. Sinus rhythm was restored in another four cases within 3–15 days after discontinuing ATRA [11, 13, 15]. Three of them required temporary pacemaker support [11, 15], and ATRA was rechallenged in two of them [15]. Interestingly, all of the incidents reported in the seven cases occurred during the induction therapy. Among the four cases with available information, three of them also experienced DS [12, 13].

The underlying mechanism of ATRA-related bradycardia has not been comprehensively studied. In 1995, Kang and Leaf demonstrated that ATRA significantly lowered the beating rate of cardiomyocytes and reduced the incidence and severity of ventricular tachyarrhythmia induced by isoproterenol in rats [16]. The inhibition of excitability may be mediated by the effect of ATRA on membrane ion channels, and this may result in conduction disturbance of the AV node [16]. Further studies are needed to elucidate the effect of ATRA on the AV node.

Another possible explanation of ATRA-related arrhythmia was leukemic cell infiltration in the myocardium. Second-degree AV block and pericardial effusion in a case with AML was reported by Hatake et al. On autopsy, focal leukemic cell infiltration in the His bundle was noted [17]. Therefore, eradication of leukemic cell could ameliorate the cardiac symptoms as reported in another case described by Civelek et al., wherein complete AV block spontaneously subsided after chemotherapy [18]. In reviewing the cases of ATRA-related arrhythmia, sinus bradycardia reported by Karakatsanis et al. also spontaneously subsided after chemotherapy without any specific intervention [14]. Moreover, all of the seven cardiac events occurred during induction chemotherapy course but not consolidation ones. These observations could possibly provide some evidence that the cardiac toxicity was partially caused by infiltration of leukemic cells in the myocardium.

As described above, APL patients receiving ATRA-based induction therapy may experience DS, which is characterized by fever, dyspnea, respiratory distress, pulmonary infiltrates, and pleural or pericardial effusions [5, 6]. The proposed pathophysiology involves tissue infiltration of APL cells. Besides infiltration of leukemic cells, ATRA also stimulates the production of chemokines and further increases lung infiltration of differentiating APL cells. Changes in cytokine production also occur in the liver, spleen, and heart [5]. According to these mechanisms, DS may also interfere with AV node conduction and result in bradyarrhythmia. In reviewing the incidents of ATRA-related bradycardia, three of four cases with available information also experienced DS [12, 13]. In Dhar's report, second-degree AV block simultaneously occurred with DS, and after steroid use, these manifestations resolved at the same time [12]. In our case, the AV block was more severe when DS occurred. Without the presentation of the DS, only mild bradycardia occurred under ATRA use. Therefore, DS may further deteriorate ATRA-related AV node conducting dysfunction. This clinical presentation strengthens the theory that DS may result in cardiac arrhythmia, which needs further study to elucidate the observation.

In conclusion, this case and literature review indicated that ATRA may cause conduction dysfunction-related arrhythmia, and it should be used more cautiously for APL patients during induction chemotherapy, especially those suffering from DS. The onset of bradycardia was usually within 1 month after initiation of ATRA. Therefore, closely monitoring vital signs and performing ECG are highly recommended during the first month of ATRA-based induction therapy. ATRA should be promptly discontinued if complete AV block occurs. In severe cases, temporary pacemaker insertion is indicated. After discontinuing ATRA, bradycardia may resolve gradually within 3–15 days. Since ATRA is the cornerstone in APL treatment, rechallenge can be considered in fully recovered and clinically stable patients.

## Figures and Tables

**Table 1:** Case reports of all-*trans* retinoic acid-associated bradycardia.

	Age	Sex	Initial WBC (mm^3^)	Indication of ATRA	ATRA dose (mg/m^2^)	Concurrent chemotherapy	Onset (days)	ECG change	Pacemaker	Response after discontinuation of ATRA	Response after rechallenge	DS
Maruhashi et al. [10]	3	M	NA	Induction	45	NA	4	Sinus bradycardia	NA	Still arrhythmia	Arrhythmia augmented	NA

Yamauchi et al. [11]	46	M	NA	Induction	45	Enocitabine	25	CAVB	TPM	NSR after 15 days	NA	NA
						Mitoxantrone						

Dhar and Barman [12]	16	F	2,000	Induction	45	NA	9	2nd degree AVB	No	ATRA was continued and steroid was given→NSR		Yes

McGregor et al. [13]	28	F	9,800	Induction	45→22.5	Idarubicin	5	Junctional bradycardia	No	NSR after 3 days	No more bradycardia	Yes

Karakatsanis et al. [14]	64	M	NA	Induction	NA	Idarubicin	8	Sinus bradycardia	No	ATRA was continued and bradycardia resolved spontaneously		NA

Shih and Wu [15]	57	M	1,600	Induction	45	NA	15	CAVB	TPM	Sinus rhythm after 4 days (under TPM)	75% dose→still AVB	No

This case	41	M	15,820	Induction	45	Idarubicin ATO	11	CAVB	TPM	HR partially recovered after interruption for 3 doses	50% dose→CAVB with DS→hold ATRA, insert TPM→1st degree AVB→25% dose→bradycardia→75% dose→NSR	Yes

ATO: arsenic trioxide; ATRA: all-*trans* retinoic acid; AVB: atrioventricular block; CAVB: complete atrioventricular block; DS: differentiation syndrome; ECG: electrocardiogram; F: female; HR: heart rate; M: male; NA: not available; NSR: normal sinus rhythm; TPM: temporary pacemaker; WBC: white blood cell.
